# Inhibitory Effect of miR-339-5p on Glioma through PTP4A1/HMGB1 Pathway

**DOI:** 10.1155/2022/2231195

**Published:** 2022-07-15

**Authors:** Buyi Zheng, Shouyi Wang, Huanan Shen, Jie Lin

**Affiliations:** ^1^Department of Neurosurgery, Wenzhou People's Hospital, Wenzhou, Zhejiang Province, China 325000; ^2^Department of Vascular & Interventional Radiology, Wenzhou People's Hospital, Wenzhou, Zhejiang Province, China 325000; ^3^Department of Neonatology, Wenzhou People's Hospital, Wenzhou, Zhejiang Province, China 325000

## Abstract

**Objective:**

Finding miR-339-5p inhibitory functions in glioma through PTP4A1/HMGB1 pathway.

**Methods:**

From May 2020 to August 2021, 20 glioblastoma and para cancer tissues were chosen for qRT-PCR analysis. The miR-NC, miR-con, miR-339-5PMIMIC, and miR-con + groups were transfected into human glioma U251 cells. The capacity of cell vascular-like structure construction was found by simulating angiogenesis, and the ability of cell movement was examined by cell scratching. The twofold luciferase reporter gene method determined that miR-339-5p targets PTP4A1, and the protein expression levels of PTP4A1 and HMGB1 were examined using Western blot.

**Results:**

MiR-339-5P expression was substantially lower in cancer samples than noncancer samples (*P* < 0.05). PTP4A1 expression in cancer samples was higher than in healthy controls (*P* < 0.05). The miR-339-5p group produced significantly less vascular-like structures than the NC and miR-con groups (*P* < 0.05). The miR-339-5p group lowered the invasive index and migratory rate of U251 cells (*P* < 0.05). PTP4A1 inhibited the luciferase activity of the pTP4A1-WT reporter gene (*P* < 0.05) but not the PTP4A1-MUT (*P* > 0.05). The miR-339-5p group had lower protein levels of PTP4A1 and HMGB1 than the NC and miR-con groups (*P* < 0.05). The development of vascular-like structures was substantially more significant in the miR-con +PTP4A1 group than in the miR-con and miR-339-5p +PTP4A1 groups (*P* < 0.05). In terms of migration and invasion index, there was a substantial difference between the miR-339-5p +PTP4A1 and the miR-con +PTP4A1 groups (*P* < 0.05). The miR-con +PTP4A1 group had a greater migration rate and invasive index than the miR-con and miR-339-5p +PTP4A1 groups (*P* < 0.05).

**Conclusion:**

MiR-339-5P inhibits angiogenic mimicry, migration, and invasion of brain glioma U251 cells by inhibiting the PTP4A1/HMGB1 signal pathway.

## 1. Introduction

Glioma is a relatively common primary intracranial tumor and is the most seen malignancy in the brain, taking up 40%-50% of the total intracranial cancers [1]. In terms of gender, the incidence of this disease is primarily male, and it is more evident in glioblastoma multiforme and medulloblastoma [2]. At present, the etiology of glioma is unknown. Glioma is a process of multifactor participation and multistep accumulation, with potential risk factors including genetic factors, specific gene polymorphism, viral infection, carcinogens of the nervous system, and ionizing radiation [3]. For patients with glioma of different grades, low-grade glioma usually has good prognoses, with a 10-year OS rate of 47% [4]. However, the prognosis is usually poor for high-grade glioma, especially for glioblastoma multiforme, with a high degree of malignancy and a 5-year OS rate of less than 3% [5]. Despite significant medical efforts in treating glioma, the prognosis of the disease remains poor. The development of glioma involves changes in various genes and cell biological behaviors. An in-depth understanding of the molecule-level causal link between the onset and developmental process of glioma can provide more accurate auxiliary diagnostic markers for the diagnoses and therapies of glioma.

The miRNAs are endogenetic noncoding RNAs playing vital regulatory roles in the onset and developmental process of tumors and are both oncogenes and tumor suppressor genes [6]. Recently, with more and more attention paid to microRNA (miRNA), it has become the focus of biological research. Relevant research has revealed that miR-339-5P is downregulated in considerably aggressive breast carcinoma and is associated with mammary carcinoma's invasive and metastatic abilities [7]. However, related reports of glioma are rare. Based on this, the present research investigated the expressing level of miR-339-5p in glioma and its molecular mechanism in glioma and its role in the PTP4A1/HMGB1 pathway, providing a valuable reference for the diagnoses and therapies of glioma.

## 2. Materials and Methods

### 2.1. qRT-PCR Identified miR-339-5P and PTP4A1 Expression in Glioma Samples

Patients diagnosed with glioma were recruited in the study. Formalin-fixed, paraffin-embedded glioma tissue blocks were obtained during surgery. The tissue samples were weighed and ground into powder in liquid nitrogen. 1.0 mL TRIZOL lysis solution (Life Technologies) was added every 20 mg of tissue powder for cracking and transferred to a centrifugal tube. Chloroform was supplemented, and the samples were left standing under ambient temperature for 5 min and afterward subjected to centrifugation at 12000 rpm at 4°C for 20 min. An equal volume of 75% ethanol was added, mixed, and centrifuged at 12000 rpm at 4°C for 10 min, the supernatant was discarded, and the RNA concentration was calculated. Total RNA was purified from liver tissue or cell cultures using the RNeasy Mini Kit (Qiagen, Chatsworth, CA) according to the manufacturer's instructions. Reverse transcription to generate cDNA was performed by using the SuperScript III First Strand Synthesis System (Invitrogen). The RNA was reversed into cDNA, and a qRT-PCR reaction was performed. The upstream of miR-339-5p was 5′ -GcacucGAGgaccucCuGUCCUS-3′, the downstream was 5′ -GGACugacauuUGagGacAGGGGA-3 ′, U6 was used as an internal reference, and the foreign body sequence was 5′-G upstream Cacucgaggaccuccuguccus-3′, downstream 5′ -GGACUGacauuUGagGACAGGGA-3′, and the reaction conditions were set as a reaction at 95°C for 5 min. Reaction at 95°C for 10s, then 60°C for the 30s, 72°C for 10 min, 40 cycles. The 2^-*ΔΔ*CT^ method was utilized for the statistic assay of the data, and the relative expression levels of target factors were obtained.

### 2.2. Cell Culture and Grouping

U251 cells (The Jackson Laboratory) were cultivated with 10% fetal bovine serum DMEM medium with 100 ng/mL streptomycin and 100 U/mL penicillin in advance and placed in an incubator with 5%CO2 at 37°C. The cells were layered onto a 50%/25% two-step Percoll gradient (Sigma) in a 50-ml conical centrifuge tube and centrifuged. After the cells were overgrown, the sterile PBS was cleaned, and 0.25% trypsin was added for digestion. Follow-up experiments were conducted when the cells were in a good growth state and the logarithmic phase. Then, they were divided into NC group, miR-con group, miR-339-5p group, and miR-339-5p +PTP4A1 group, and transfection reagent Lipofectamine 3000 was applied. miR-NC was transfected into U251 as NC group, miR-con was transfected into U251 as a miR-con group, and miR-339-5p was transfected. Mimic (Qiagen, Chatsworth, CA) was transfected into U251 as a miR-339-5p group, miR-con (Qiagen, Chatsworth, CA) and PCDNA3.1-PTP4A1 (Qiagen, Chatsworth, CA) were transfected into U251 as miR-con +PTP4A1 group, and miR-339-5p was transfected. Mimic and PCDNA3.1-PTP4A1 were transfected into U251 as miR-339-5p +PTP4A1 group, respectively.

### 2.3. Angiogenesis Mimicry Was Used to Detect Lumen Formation

The transfected brain glioma cells U251 were digested with tryptic enzyme and cultured for 48 h. BD matrix glue was laid on a precooled 24-well plate with 400 *μ*l per well and stood for 30 min before solidification. The trypsin digested U251 cells of every group were placed in a 24-well dish with 1.0 × 10^5^ cells/well. After 24 h culture, the confocal microscope was used to record the results. Five fields were randomly selected under a high-power microscope to record the number of lumens formed by the cells.

### 2.4. The Migration of U251 Cells Was Detected by Cell Scratch Assay

The transfected brain glioma cells U251 were inoculated with 1 × 10^5^ cells/well in 6-well dishes and cultivated under ambient temperature for 12 h to observe the cell growth. When the degree of cell fusion reached 80%, a line was gently drawn at the bottom of each plate with a 20 *μ*l spear tip. The culture was continued at 37°C in a 5%CO2 constant temperature incubator, and photos were taken under an inverted microscope to record the cell growth. Mark the width.

### 2.5. Transwell Detected U251 Cell Invasion

Matrigel matrix glue was dissolved overnight at 4°C, mixed with serum-free DMEM culture medium, and then spread on polycarbonate membrane in the Transwell chamber. Matrigel was polymerized into a gel, digested by trypsin 24 h later, and the cellular content was modified to 10^5^ cells/mL. The cellular suspension was transferred to the upper chamber of Transwell and incubated for 48 h. After that, it was subjected to fixation in methyl alcohol, dyed in 0.1% gentian violet for 20 min, and cleaned in PBS. Five fields were stochastically chosen and counted under an inverted microscopic device, and photographed.

### 2.6. Testing the miR-339-5p-PTP4A1 Targeting Relationship with Dual-Luciferase Reporter Genes

Each well was covered with about 3 × 10^4^ U251 cells in 24-well plates and incubated in an incubator for about 24 h. Then, according to the instructions of the Lipofectamine 3000 reagent, the double luciferase reporter plasmid, miR-NC, and miR-339-5p mimic were transferred into U251 cells. After 48 h transfection, double luciferase was used. The transfected U251 cells were lysed by the report system, and fluorescence values were detected by the BioTEKSynergy2 multifunctional microplate analyzer to identify the adsorption of PTP4A1 on miR-339-5p.

### 2.7. Western Blot Identified PTP4A1 and HMGB1 Protein Expression

Cells in every group were harvested and lysed. Protein quantification was performed using the BCA protein quantification kit. SDS-page gel electrophoresis was performed, and the membrane was transformed. HRP labeled antibody was added (Abcam, ab9485, diluted at 1 : 300) and incubated for 30 min. ECL luminescent solution was uniformly added to the PVDF membrane. Tecon exposure machine was exposed, and ImageJ software was used to analyze gray values.

### 2.8. Statistical Treatment

SPSS21.0 program was utilized for processing, and measured data was represented by $\left(\bar{x}\pm s\right)$. T-check was used for inter-group data comparison, and variance analysis was utilized for data contrast amongst several groups; *P* < 0.05 had significance in statistics.

## 3. Results

### 3.1. Glioma miR-339-5p and PTP4A1 Expression

**Figure 1:**
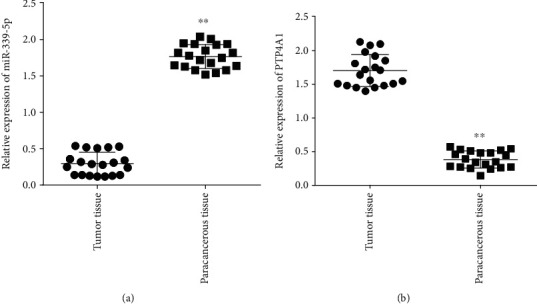
MiR-339-5p and PTP4A1 expression in glioma tumors and normal tissues. Comparative expression of Mir-339-5p and PTP4A1, ∗∗*P* < 0.01.

Rt-qPCR results revealed that the expressing level miR-339-5P in cancer samples was considerably lower than neighboring nontumor specimens (*P* < 0.05), as presented in Figure 1(a). PTP4A1 expressing level in tumor tissues was considerably higher vs. neighboring specimens (*P* < 0.05), as presented in Figure 1(b).

### 3.2. Effects of miR-339-5P on Angiogenic Mimicry in Glioma Cells

**Figure 2:**
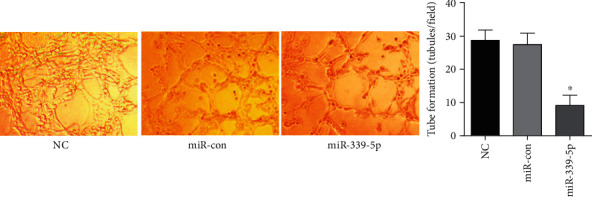
The involvement of miR-339-5p in glioma angiogenic mimicry, ∗*P* < 0.05.

24h later, it was found that the U251 cells of NC group, miR-con group , and the miR-339-5p group fused and connected to produce a blood vessel-shaped architecture. In contrast to the NC and the miR-con groups, the forming of such architecture within the miR-339-5p group was considerably decreased (*P* < 0.05), as presented in Figure 2.

### 3.3. Role of miR-339-5P in Migratory and Invasive Abilities of Glioma Cells

**Figure 3:**
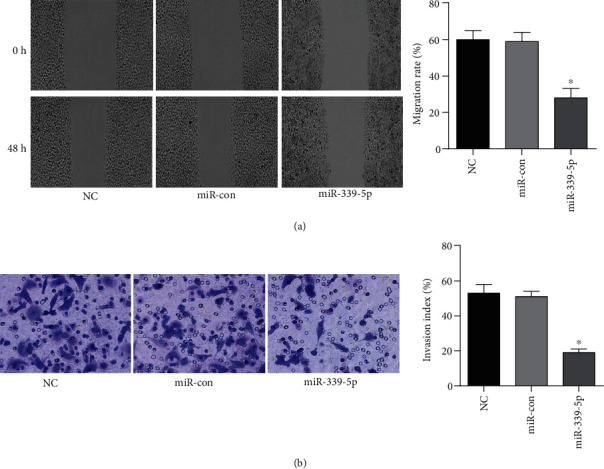
The involvement of miR-339-5p in glioma cell migration and invasion. All group cells migrate; all group cells invade, ∗*P* < 0.05.

The migration and invasion indices of GLIoma U251 cells did not change substantially between the NC and miR-con groups (*P* > 0.05). In contrast, GLIoma U251 cells in the miR-339-5p group had a lower migratory rate and invasion index (*P* < 0.05), as presented in Figure 3.

### 3.4. Targeting Association between miR-339-5p and PTP4A1

**Figure 4:**
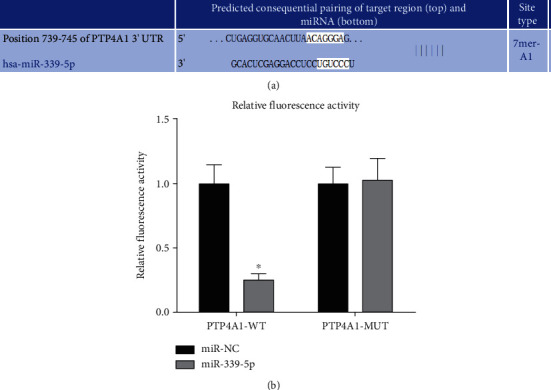
Bioinformatics analysis of dual-luciferase reporter gene to verify that miR-497-5p targets PTP4A1. (a) Binding spot of PTP4A1 3′-UTR to Mir-339-5p. (b) Comparative fluorescence activity, ∗*P* < 0.05.

The 3′-UTR region of PTP4A1 had a target binding location of miR-339-5p, according to the TargetScan website. As shown in Figure 4, miR-339-5p significantly reduced the luciferase activity of the pTP4A1-WT reporter gene compared to the miR-NC group (*P* < 0.05) but did not affect PTP4A1-MUT (*P* > 0.05).

### 3.5. Role of miR-339-5P in PTP4A1/HMGB1 Pathway

**Figure 5:**
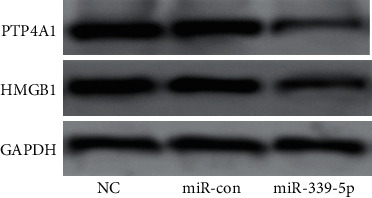
The roles of miR-339-5p in the expressing level of PTP4A1 and HMGB1 proteins.

Western blot showed that no remarkable diversity existed in the expression of PTP4A1 and HMGB1 between the NC and miR-con groups (*P* > 0.05); in contrast to the NC and miR-con groups, the protein-expressing levels of PTP4A1 and HMGB1 in the miR-339-5p group was considerably diminished (*P* < 0.05), as presented by Figure 5.

### 3.6. Role of miR-339-5p Targeting PTP4A1/HMGB1 Pathway in Angiogenic Mimicry in Glioma Cells

**Figure 6:**
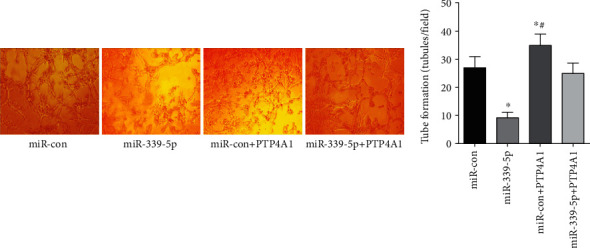
The mimicry of angiogenesis of glioma cells in each group, ∗*P* < 0.05 (compared with miR-con group), ^#^*P* < 0.05 (compared with miR-339-5p+PTP4A1).

24 h later, the vascular-like structure in the miR-339-5p group was considerably lower vs. the miR-con group, miR-con+PTP4A1 group, and miR-339-5p+PTP4A1 group (*P* < 0.05), the formation of vascular-like structure in miR-con +PTP4A1 group was considerably higher vs. miR-con and miR-339-5p+PTP4A1 groups (*P* < 0.05), and no remarkable diversity existed in vascular structure between the miR-con and miR-339-5p +PTP4A1 groups (*P* > 0.05), as presented in Figure 6.

### 3.7. Roles of miR-339-5P Targeting the PTP4A1/HMGB1 Pathway in the Metastasis and Aggression Capabilities of Glioma Cells

**Figure 7:**
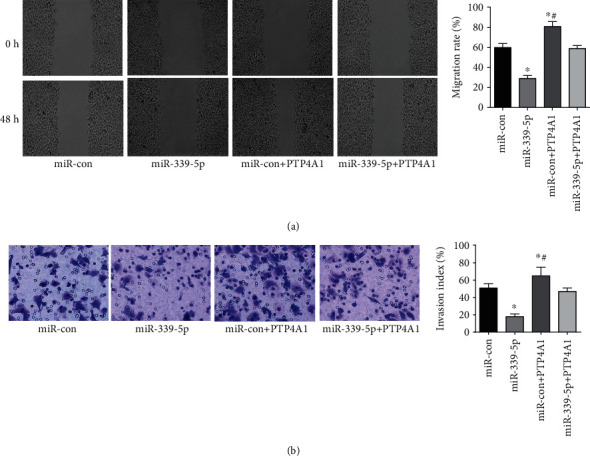
Metastasis and aggression capabilities of glioma cells in every group. (a) Migration of cells in every group. (b) The invasion of every group of cells, ∗*P* < 0.05 (compared with miR-con group), ^#^*P* < 0.05 (compared with miR-339-5p+PTP4A1).

The miR-339-5p group had a lower metastasis rate and aggression index than the miR-con, miR-con+PTP4A1, and miR-339-5p+PTP4A1 groups (*P* < 0.05), while the miR-con+PTP4A1 group had a higher migratory rate and aggression index than the miR-con and miR-339-5p+PTP4A1 groups (*P* < 0.05). Unlike the miR-339-5p+PTP4A1 group, the glioma U251 cells' migratory rate and invasive index were not significantly different (*P* > 0.05), as presented in Figure 7.

## 4. Discussion

Glioma is a commonly seen kind of malignancy in the CNS, induced via the cancerization of glial cells in the cerebrum and CNS [8]. The parietal lobe, temporal lobe, and frontal lobe are the most common disease sites, which are featured by high prevalence, relapse rate, and death rate [9]. In recent years, the incidence of primary glioma has been increasing year by year, and the average survival time of surgery combined with radiotherapy and chemotherapy is only 8-11 months [10]. The main clinical manifestations of glioma are global cranial or localized high cranial pressure, and different tumor locations determine the neurological deficit [11]. Currently, glioma therapy is still predominantly surgical resection, and attention should be paid to minimizing the surgical injury as much as possible [12]. Important neural structures such as the brainstem and ventricle are involved for those that cannot be surgically removed. Ensuring the integrity of neural structures is particularly important for improving the prognosis of patients. Local radiotherapy, chemotherapy, and gene therapy can be used for a few palliative tumor tissues after surgery [13]. Gene therapy is the leading investment direction of the scientific community at present, and the treatment methods are gradually maturing; the operation cost is constantly reduced, the feasibility is constantly improved, and it will be likely to become a good choice for treating glioma.

MiRNAs are endogenous noncoding small molecule RNAs that impede and degrade mRNA translation [14]. MiRNAs may also initiate transcription, bind Toll-like receptors, and control protein expression [15]. MiRNAs are possible novel therapeutic targets and biomarkers for cancer [16]. Studies have shown that miRNA regulates glioma cell genesis, development, proliferation, invasion, and metastasis [17]. MiR-339-5P expression is linked to lymphatic metastases and may inhibit mammary cancer cell proliferation, invasion, and metastatic potential [18]. This study validated the presence of miR-339-5p in glioma samples and its role in vascular structure creation, migration, and invasion in brain glioma U251 cells. The findings suggest that low levels of miR-339-5P expression in glioma tissues may limit vascular structure growth, migration, and invasion. The regulation of miR-339-5P expression in lung cancer cells has been demonstrated to impede cell growth and induce programmed cell death, similar to our findings [19].

TargetScan predicted the targeting link between miR-339-5p and PTP4A1 in this investigation. The findings demonstrated that miR-339-5p reduced the luciferase activity of PTP4A1-WT and that PTP4A1 expression was increased in glioma samples. PTP4A1 is a family member of liver cell regeneration phosphatase factor proteins, which plays an essential role in tumor development [20]. Further studies have shown that the expression of PTP4A1 is high in many malignancies and their cell lines, which can facilitate oncocyte aggression and migration [21]. The level of PTP4A1 expression affects the degree of malignancy of tumors and the malignant biological behavior of cancer cells [22, 23]. HMGB1 is a nuclear protein that initiates inflammation through Toll-like receptors or advanced glycation products and induces the invasion and migration of malignant tumor cells [24, 25]. The findings demonstrated that overexpression of miR-339-5p might reduce PTP4A1 and HMGB1 protein expression. To confirm the targeting relationship between miR-339-5p and PTP4A1, miR-339-5p will be transfected. Following PCDNA3.1-PTP4A1 transfection, the formation of vascular-like structure, migration rate, and invasion index in the miR-339-5p+PTP4A1 group was considerably greater vs. miR-339-5p group and smaller than the miR-con+PTP4A1 group, indicating that U251 cells that were overexpressing miR-339-5p can target the expressing of PTP4A1, further reduce the expression of HMGB1 protein, and inhibit its vascular-like structure formation, cellular migratory, and invasive abilities.

To sum up, mir-339-5p is downregulated in glioma specimens, and overly expressed miR-339-5p can repress the vascular-like structure formation and cellular migratory and invasive abilities of U251 cells. The process may be linked to reduced PTP4A1 expression and HMGB1 levels.

The current study has several limitations. We attempted to explore the miR-339-5p inhibitory functions in glioma and observe the underlying mechanism miR-339-5p involved in. However, this study lacks the validation of mouse tumor bearing model. In turn, this may limit the interpretation of the effect of miR-339-5p inhibitory functions in glioma. In future research, we will continue to supplement research in the related fields.

## Data Availability

The data used to support the findings of this study are available from the corresponding author upon request.
